# Application of a novel index for understanding vascular health following pharmacological intervention in a pre-clinical model of metabolic disease

**DOI:** 10.3389/fphar.2023.1104568

**Published:** 2023-01-25

**Authors:** Nithin J. Menon, Brayden D. Halvorson, Gabrielle H. Alimorad, Jefferson C. Frisbee, Daniel J. Lizotte, Aaron D. Ward, Daniel Goldman, Paul D. Chantler, Stephanie J. Frisbee

**Affiliations:** ^1^ Department of Medical Biophysics, London, ON, Canada; ^2^ Department of Epidemiology and Biostatistics, London, ON, Canada; ^3^ Department of Computer Science, Faculty of Science, University of Western Ontario, London, ON, Canada; ^4^ Lawson Health Research Institute, London, ON, Canada; ^5^ Department of Human Performance-Exercise Physiology, School of Medicine, West Virginia University, Morgantown, WV, United States; ^6^ Department of Pathology and Laboratory Medicine, Schulich School of Medicine and Dentistry, University of Western Ontario, London, ON, Canada

**Keywords:** vascular dysfunction, vascular disease, rodent models of metabolic disease, data analytics, vascular health outcomes

## Abstract

While a thorough understanding of microvascular function in health and how it becomes compromised with progression of disease risk is critical for developing effective therapeutic interventions, our ability to accurately assess the beneficial impact of pharmacological interventions to improve outcomes is vital. Here we introduce a novel Vascular Health Index (VHI) that allows for simultaneous assessment of changes to vascular reactivity/endothelial function, vascular wall mechanics and microvessel density within cerebral and skeletal muscle vascular networks with progression of metabolic disease in obese Zucker rats (OZR); under control conditions and following pharmacological interventions of clinical relevance. Outcomes are compared to “healthy” conditions in lean Zucker rats. We detail the calculation of vascular health index, full assessments of validity, and describe progressive changes to vascular health index over the development of metabolic disease in obese Zucker rats. Further, we detail the improvement to cerebral and skeletal muscle vascular health index following chronic treatment of obese Zucker rats with anti-hypertensive (15%–52% for skeletal muscle vascular health index; 12%–48% for cerebral vascular health index; *p* < 0.05 for both), anti-dyslipidemic (13%–48% for skeletal muscle vascular health index; *p* < 0.05), anti-diabetic (12%–32% for cerebral vascular health index; *p* < 0.05) and anti-oxidant/inflammation (41%–64% for skeletal muscle vascular health index; 29%–42% for cerebral vascular health index; *p* < 0.05 for both) drugs. The results present the effectiveness of mechanistically diverse interventions to improve cerebral or skeletal muscle vascular health index in obese Zucker rats and provide insight into the superiority of some pharmacological agents despite similar effectiveness in terms of impact on intended targets. In addition, we demonstrate the utility of including a wider, more integrative approach to the study of microvasculopathy under settings of elevated disease risk and following pharmacological intervention. A major benefit of integrating vascular health index is an increased understanding of the development, timing and efficacy of interventions through greater insight into integrated microvascular function in combination with individual, higher resolution metrics.

## Introduction

The role and relevance of microvascular dysfunction as a significant contributor to the functional or clinical outcomes under the broader umbrella of cardio- or cerebrovascular disease risk has long been an area of great focus in both basic and translational research. However, although many diverse efforts have been made to investigate individual correlative measures and predictive biomarkers of vascular dysfunction, far fewer have attempted to understand and frame integrated vascular function within the context of health, disease, and treatment (Martins et al., 2021; Amal et al., 2022).

We identify three key challenges that impede a more integrated analysis of vascular function. First, despite expanded insight into the emergence and impact of metabolic diseases on health outcomes, the interpretation of data can be complicated as a result of the multi-variate nature of not only microvascular structure and function, but the temporal development of these adaptations to disease risk. Second, although they are powerful in terms of understanding the diversity of disease and in facilitating investigations evaluating the broader relative dysfunctional states of the vasculature in various disease states and pharmacological treatment regimes, heterogeneity and diversity in models of vasculopathy that are used further complicate efforts to integrate datasets and thereby to produce opportunities for more detailed, powerful advanced analytic approaches. Third, basic science investigators face challenges when integrating results across multiple laboratories because differences in goals, methodology and expertise may not allow facile merging of datasets.

Given that the overwhelming majority of individual research efforts have a complex study design with highly specialized protocols/preparations chosen to support or refute a specific hypothesis or question, it can be problematic to use resulting data beyond the initial intent of each study. This hinders our ability to create integrated secondary data sources from which results can be pooled for greater analytic and inferential power, and profoundly limits our ability to access a variety of advanced analytic approaches including machine learning, artificial intelligence and metanalyses. In order to access such advanced techniques, basic scientists must have approaches that allow for the pooling of datasets across multiple primary studies across levels of spatial and temporal resolution that facilitate data interpretation from a broader construct of “health” and “disease.”

In response to these myriad challenges, we develop and describe a standardized, integrated measure of vascular health and dysfunction (Menon et al., 2022). The Vascular Health Index (VHI) allows for the simultaneous assessment of changes to vascular reactivity/endothelial function, vascular wall mechanics, and microvessel density within skeletal muscle and cerebral vascular networks with the progression of chronic metabolic disease. This VHI will quantify vascular dysfunction in states of elevated peripheral vascular disease and cerebrovascular disease risk relative to the vascular function of healthy, age-matched control animals (Menon et al., 2022). Further, the present study also focuses on the impact of clinically relevant pharmacological interventions to change VHI from untreated control conditions and can provide some insight as to why some interventions have more effective functional outcomes despite comparable impacts on specific risk factors. The impact of these pharmacologic interventions on the integrated vascular function of a network are of great interest in both the development and evaluation of prodromic and postdromic intervention targeting to achieve ideal health outcomes.

## Materials and methods

The majority of the data presented here have been published previously and these citations will be made at the appropriate and relevant points within the text. The present manuscript also represents the inclusion of *de novo* experiments/analyses, previously unpublished results/analyses, and the integration of data from previous studies for novel analyses. The protocols and specific methodology for the collection of the specific vascular phenotypes and the subsequent calculation of the Vascular Health Index (VHI) are detailed and referenced below.

### Animal model

All experiments and analyses described in this manuscript use male lean (LZR) and obese (OZR) Zucker rats. Animals were purchased from the supplier (Harlan/Envigo) at 6–7 weeks of age and were housed in an accredited animal care facility at the Medical College of Wisconsin, West Virginia University, or the University of Western Ontario, with *ad libitum* access to normal chow and water until the time of final usage unless otherwise noted. Following 1 week of acclimation, rats were placed into one of the following groups until their final usage.

1. Time control (LZR and OZR without intervention and aged to a maximum of ∼20 weeks).
2. Anti-hypertensive groups:
  a) OZR treated with captopril [angiotensin converting enzyme inhibitor; 60 mg·kg^−1^ day^−1^; mixed with food (Frisbee, 2005a; Frisbee et al., 2018)].
  b) OZR treated with hydralazine [smooth muscle hyperpolarizer/vasodilator; 50 mg·kg^−1^ day^−1^; mixed with food (Frisbee, 2005a; Frisbee et al., 2018)].
3. Anti-dyslipidemia groups (skeletal muscle VHI only):
  a) OZR treated with atorvastatin (HMG Co-A [3-hydroxy-3-methyl-glutaryl-coenzyme A reductase] reductase inhibitor); 25 mg·kg^−1^ day^−1^; mixed with food (Goodwill et al., 2009; Frisbee et al., 2018).
  b) OZR treated with gemfibrozil (peroxisome proliferator-activated receptor-α activator); 100 mg·kg^−1^ day^−1^; mixed with food (Goodwill et al., 2009; Frisbee et al., 2018).
4. Anti-diabetes groups (cerebral VHI only):
  a) OZR treated with metformin [hepatic gluconeogenesis inhibitor; 300 mg·kg^−1^ day^−1^; drinking water (Chantler et al., 2015; Frisbee et al., 2018)].
  b) OZR treated with rosiglitazone [insulin sensitizing agent; 10 mg·kg^−1^ day^−1^; mixed with food (Chantler et al., 2015; Frisbee et al., 2018)].
5. Antioxidant/Anti-inflammatory/Nitric oxide bioavailability groups:
  a) OZR treated with TEMPOL (antioxidant); 10^−3^ mol/L day^−1^; mixed in drinking water (Frisbee et al., 2014; Frisbee et al., 2018).
  b) OZR treated with pentoxifylline [skeletal muscle VHI only; inhibition of tumour necrosis factor alpha (TNF-α) production; 30 mg·kg^−1^ day^−1^; i.p., injection (Frisbee et al., 2018)].
  c) OZR treated with L-NAME (L-N^G^-Nitro arginine methyl ester; non-selective nitric oxide synthase inhibitor); 10^−4^ M, in drinking water (Frisbee et al., 2007; Frisbee et al., 2018)].

Throughout the treatment of rats with the agents listed above, changes in body mass as well as daily food and water consumption with age were taken into account to maintain proper dosing as well as changes to circulating blood volume (Frisbee, 2005b; Goodwill et al., 2009; Frisbee et al., 2018).

At the time of final usage, each rat was deeply anesthetized with sodium pentobarbital (50 mg·kg^−1^ i.p.) and the trachea was intubated to maintain a patent airway. In all rats, a carotid artery and an external jugular vein were cannulated to measure arterial pressure and to infuse additional anesthetic, respectively, as necessary. At this time, an aliquot of blood was drawn from the jugular vein of each animal to be used for the subsequent determination of plasma metabolic/endocrine, oxidant stress, and inflammatory biomarker profiles using commercially available kits (Frisbee et al., 2018). All procedures followed approved IACUC protocols at each institution.

### Evaluation of vascular reactivity

The assessment of arteriolar reactivity from skeletal muscle was determined using the intramuscular continuation of the gracilis arteries, which were removed from each leg following the procedures in the neck (above). Subsequently, the rat was given a lethal overdose of pentobarbital anesthetic, followed by the removal of the head *via* decapitation. For the assessment of cerebrovascular reactivity, the middle cerebral arteries (MCA) were removed from their origin on the Circle of Willis following the removal of the brain from the skull. Both the gracilis muscle arterioles and the MCAs were doubly-cannulated and placed in a heated chamber (37°C) that allowed the vessel lumen and exterior to be perfused and superfused, respectively, with physiological salt solution (PSS; equilibrated with 21% O_2_, 5% CO_2_; 74% N_2_) from separate reservoirs (Fredricks et al., 1994a; Liu et al., 1997). Vessel diameter was measured using television microscopy and an on-screen video micrometer. Both vessels were extended to their *in situ* length and were equilibrated at 80% of the animal’s mean arterial pressure (Fredricks et al., 1994b; Liu et al., 1997).

In both gracilis muscle arterioles and MCAs, vascular reactivity was evaluated in response to application of increasing concentrations of acetylcholine (10^−9^ M–10^−6^ M) in order to assess endothelial function and dilator responses (Liu et al., 1997; Halvorson et al., 2021).

The mechanical responses of isolated arterioles following pharmacological challenge with any of the agonists were fit with the following logistic equation:

$$y=\min+\left[\frac{\max-\min}{1+10^{\log EC_{50}-x}}\right]$$

where y represents the change in arteriolar diameter, “min” and “max” represent the lower and upper bounds, respectively, of the change in arteriolar diameter with increasing acetylcholine concentration, x is the logarithm of acetylcholine concentration and $\log EC_{50}$ represents the logarithm of acetylcholine concentration ( x ) at which the response ( y ) is halfway between the lower and upper bounds.

### Evaluation of vascular wall mechanics

Following the experimental procedures for measuring *ex vivo* vascular reactivity for both MCA and gracilis arterioles, the perfusate and superfusate PSS were replaced with Ca^2+^-free PSS containing the metal ion chelators EDTA (0.03 mM) and EGTA (2.0 mM). Vessels were challenged with 10^−7^ M phenylephrine (gracilis arterioles) or serotonin (middle cerebral arteries) until all active tone was lost. Subsequently, intralumenal pressure within the isolated vessel was altered, in 20 mmHg increments, between 0 and 160 mmHg. To ensure that a negative intralumenal pressure was not exerted on the vessel, 5 mmHg was used as the “0 mmHg” intralumenal pressure point; all other values of intralumenal pressure were multiples of 20 mmHg up to 160 mmHg. After ∼5 min at each intralumenal pressure, the inner and outer diameter of the isolated vessel was determined.

All calculations of arteriolar wall mechanics (used as indicators of structural alterations to the individual microvessel) are based on those used previously (Baumbach and Hajdu, 1993; Brooks et al., 2015). The resulting stress versus strain relationship from each vessel was fit (ordinary least squares analyses, *r* ^2^ > 0.85) with an exponential growth equation, where higher levels of slope (*β*) are indicative of increasing arterial stiffness [i.e., requiring a greater degree of distending pressure to achieve a given level of wall deformation (Milnor, 1982; Baumbach and Hajdu, 1993)].

### Evaluation of skeletal muscle microvessel density

From each rat, the gastrocnemius muscle from the left leg was removed, rinsed in PSS and fixed in 0.25% formalin. Muscles were embedded in paraffin and cut into 5 μm cross sections. Sections were incubated with *Griffonia simplicifolia* I lectin (GS-1; a general microvessel stain for all vessel <20 μm diameter; (Greene et al., 1990; Hansen-Smith et al., 1990), for subsequent determination of microvessel density using immunohistochemistry and fluorescence microscopy for the microvessel counting procedures (Frisbee, 2003; Frisbee et al., 2014).

### Determination of cerebral cortex microvessel density

Following removal of the MCAs from the Circle of Willis on the base of the brain, the brain was placed within Tissue-Tek OCT compound and frozen. Brains were then sliced into 5 μm cross sections and where then stained using the established approach developed previously using primary anti-CD-31 antibody (Munzenmaier and Greene, 2006; Chantler et al., 2015). Under microscopy, localization of labeled microvessels and subsequent microvessel counting procedures were done as described previously (Chantler et al., 2015).

### Determination of vascular health index (VHI) characteristics

In developing the VHI and ensuring it can effectively capture critical aspects of vascular structure and function, the following fundamental considerations were made.

1) Given the many structural and functional differences exist between the cerebral and skeletal muscle vasculature, a metric representing the health of their vasculature must be calibrated and calculated separately to yield a cerebral VHI and a peripheral VHI.
2) The metric must be a composite measure that accounts for distinct and relevant aspects of vascular function and structure.
3) The metric will not be a predictive measure but instead will describe the relative state of the vasculature at a given time. Given this, there is no *a priori* basis for establishing parameter weighting or coefficients for the different components within the composite metric. That is, all components of the VHI are given equal weighting.
4) The use and calculation of such a metric needs to be practical and feasible. As such, the components of the metric need to be relatively easily collected in sufficient frequency to facilitate the actual quantitative determination of the metric in significant amounts, not just for our research group but other interested research teams as well.
5) Given that the validity of a metric is the degree to which values from that metric represent the variable they intend to, we will need several forms of evidence to establish VHI as a valid estimator of vascular health. The specific forms of evidence/aspects of validity of concern are:
  a) Face validity (Price et al., 2015): the extent to which a metric appears to measure the construct of interest; are the parameters used in the development of the metric appropriate to the intention?
  b) Content Validity (Price et al., 2015): determines whether the index is appropriately representative of the aspects of the system being modeled. Does the content of the metric encompass the relevant aspects it is intended to estimate?
  c) Criterion Validity (Price et al., 2015): the extent to which the index responds in a manner that is consistent with general understanding and developed hypotheses and represents how well the value of the metric is indicative of the underlying theory of the system. Specifically, it is the extent to which values of a metric are correlated with other criterion variables with which one would expect the measure to be correlated.
  d) Discriminant Validity (Price et al., 2015): the ability of the metric to distinguish between cohorts with differing levels of the underlying construct (in this case, vascular health). For example, VHI must be able to distinguish a cohort of OZR from LZR as well as any effective treatment group that works to mitigate or minimize vascular dysfunction (e.g., a population of OZR receiving a therapeutic pharmacological treatment).

In evaluating the above criteria, we developed a three–component calculation for of both cerebral and peripheral VHI in LZR and OZR. The use of a three–component metric (comprised of readily collected measures) allows for larger sample sizes to be assessed while still capturing the essential aspects of vascular health, allowing for a more facile, easily integrated and broadly applicable approach for investigators (Menon et al., 2022). The inclusion of additional components to the calculation of VHI will, by definition, make the determination more complicated, requiring more extensive and diverse data collection, with the potential introduction of greater error and variability, potentially confounding interpretation. The initial development of the VHI was constructed using a different dataset from that for the present study (i.e., VHI was not specifically constructed for application to the treatment effects in the current dataset) (Menon et al., 2022).

### VHI parameter selection

Three fundamental aspects of healthy vasculature are the ability of resistance vessels to respond appropriately to vasoactive stimuli, the mechanics (i.e., the distensibility or stiffness) of the arteriolar wall, and the structure of the microvascular network from the perspective of a microvessel/capillary density within perfused tissue (Effros et al., 1981). Thus, to ensure face validity as well as content validity, the components of the VHI were selected to represent these differing major descriptors of vascular health.

### Assessing the four aspects of validity

#### Face validity

**FIGURE 1:**
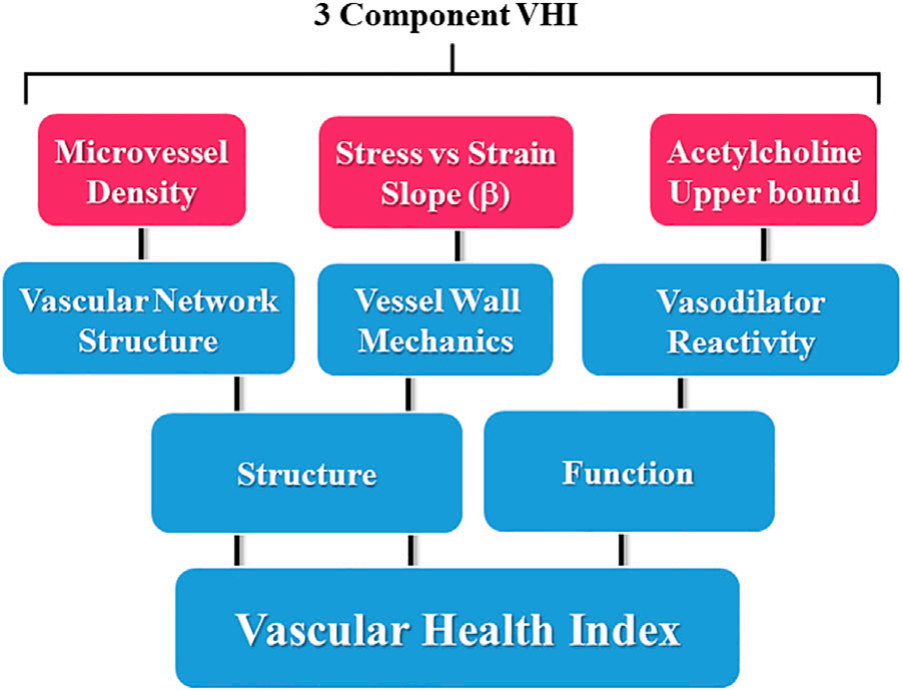
Schematic representation of the components contributing to the three-parameter calculations for the Vascular Health Index (VHI), and their context within the scope of vascular structure and function. Please see text for details.

Being the weakest and least rigorous form of validity, face validity is often assessed informally. For our purposes, the assessment was made in the process of parameter selection for the components of the measure. This is represented in Figure 1, where the conceptual design summarizes the major aspects of vascular function used in the present study, where each of the domains are represented in the calculations of VHI.

#### Content validity

The content validity of the measure was ensured by clearly defining and restricting the components of “vascular health” to three major aspects and appropriately representing those aspects in the VHI. The three main aspects of vascular health are the reactivity of resistance vessels, arteriolar wall mechanics, and microvessel density within the tissue (Effros et al., 1981).

#### Criterion validity

In assessing criterion validity, we need to identify a variable with which we would expect individual values of VHI in a given population to be correlated. For the present study, both plasma insulin and TNF-α concentrations were selected as the criterion with an expected correlation to VHI in a population of obese Zucker rats, given the well-established demonstration of changes in their circulating concentrations with increasing severity and duration of metabolic disease (Ebenezer et al., 2009; Burgmaier et al., 2010). To demonstrate criterion validity, there must be a strong correlation between plasma insulin and TNF-α concentrations and VHI in OZR through the age range of the study.

#### Discriminant validity

We will demonstrate the metric’s discriminant validity by distinguishing specific populations of Zucker rats using only their VHI values. Specifically, cohorts of OZR that have been treated with a blocker of nitric oxide bioavailability (L-NAME) to accelerate the degradation of the three measures of vasculopathy used in the present study (Frisbee, 2005c; Frisbee et al., 2014).

#### Construction of the measure

The construction of the VHI was done using data that were distinct from that in the present study with no overlap. This is an important consideration as it removes potential bias from any attempt to produce results and interpretations specific to the current data set. In constructing and calculating VHI, the following principles were used:

1. The metric will be calculated at different age points (7, 10, 13, 17, and 20 weeks old). This allows for comparisons between lean and obese Zucker rats across all ages of the animal and the resultant composite score can be considered to be independent of the specific age.
2. With “ideal” vascular health quantified in LZR, an animal experiencing an altered condition from this (e.g., elevated disease risk, interventional treatments) can then be quantitatively compared to this ideal standard. The most direct mechanism for accomplishing this is to calculate a percentage-based score, where the measurement for the “sick” animal is expressed as a percentage (%) of the value for the age-matched healthy standard. Thus, the VHI metric is interpreted as % of ideal vascular health.
3. Within each of the three components comprising VHI, the percentage scores are converted to a percentile rank. This step is taken to normalize the differing variability or ranges of values that can be demonstrated in each of the three VHI components due to scale and the (patho) physiologic range of results. VHI is meant to weigh all components equally and was not artificially dominated by any single component. This was necessary for the components used in the present study, as alterations in dilator reactivity and microvessel density in either the skeletal muscle or cerebral circulation are invariably modest as compared to changes in the slope (*β*) coefficient describing alterations to vascular wall mechanics in OZR, which can frequently be multiples of the values determine in LZR. The use of percentile ranking prevents the changes in wall mechanics from dominating the calculation of VHI. VHI is then calculated by averaging the component score (% of ideal) across three components in the index; repeated for both the cerebral and peripheral VHI.
4. To determine the index in control LZR, calculations were performed as outlined in Table 1. It should be noted that, with both age and disease risk, some variables are expected to increase as animals become unhealthy (e.g., vascular wall stiffness) whereas others are expected to decrease (e.g., endothelial function). As shown in Table 1, this has been addressed in the calculations for the vascular wall stiffness component score by treating the relative increase above the associated standard of health value as a corresponding deficit to the component score (%).
5. The metric will be a measure of “health,” where untreated LZR that served as control animals in previous studies were used to define ideal vascular function (i.e., ideal standards for each component of VHI) at different age points. Therefore, the VHI of a given animal will represent any unhealthy deviation from normal in its vasculature. Given the use of data across multiple studies, the VHI of any age-matched animal under an experimental condition will be calculated relative to the specific control values (i.e., untreated LZR) from the original experiment.

**TABLE 1:** Calculations used for the determination of individual VHI Components in the present study.

Component	Expected deviation from LZR with disease risk	Formula used to calculate VHI component
Acetylcholine-induced dilation (upper bound; μm)	Reduced (↓)	$\frac{Measurement}{Standard\;of\;health}\times 100$
Microvessel density (#/mm^2^)	Reduced (↓)	$\frac{Measurement}{Standard\;of\;health}\times 100$
Circumferential stress vs. strain slope coefficient (*β*)	Increased (↑)	$100+\left(100-\left[\frac{Measurement}{Standard\;of\;health}\times 100\right]\right)$

### Statistical analyses

Significant differences in baseline characteristics (Table 3), skeletal VHI components (Table 4), and cerebral VHI components (Table 5) across groups were analyzed with analysis of variance (ANOVA) or repeated measures ANOVA, as appropriate, followed by Newman-Keuls *post hoc* test to determine differences between specific groups. Pearson correlation coefficients between VHI and plasma insulin or VHI and TNF-α were calculated and used to demonstrate the association between parameters and demonstrate criterion validity.

## Results

Table 2 summarizes the samples sizes of all animal groups, at all ages, used in the present study. The baseline characteristics of the animals used in the present study, at each age, are summarized in Table 3. Tables 4, 5 present the raw data describing the different VHI components used in the present study for the peripheral (skeletal muscle) and cerebral vasculature, respectively.

**TABLE 2:** Animal numbers used in the present study. Data are presented for each group of animals and at each age for both the peripheral and cerebral vascular health index (VHI) calculations.

	Age (Wks)	LZR	OZR	OZR + HYD	OZR + CAP	OZR + GEM	OZR + ATOR	OZR + PEN	OZR + MET	OZR + ROSI	OZR + TEM	OZR + LNM
Skeletal muscle VHI	7	51	36	4	10	10	10	4	—	—	4	4
	10	37	30	4	10	10	10	4	—	—	4	4
	13	44	36	5	10	10	10	—	—	—	4	4
	17	48	28	4	10	10	10	4	—	—	9	4
	20	6	6	—	—	—	—	—	—	—	—	—
Cerebral VHI	7	36	20	6	6	—	—	—	6	6	6	6
	10	18	8	—	—	—	—	—	—	—	—	—
	13	24	14	6	6	—	—	—	6	6	6	6
	17	18	12	6	6	—	—	—	6	6	6	6
	20	19	8	—	—	—	—	—	—	—	—	—

**TABLE 3:** Baseline characteristics of animals used in the present study.

Variable	Group	7 wk	10 wk	13 wk	17 wk	20 wk
Mass (g)	LZR	149.7 ± 1.5^†^	243.0 ± 2.1^†^	307.2 ± 2.0†	357.5 ± 1.5^†^	374.3 ± 2.6^†^
	OZR	233.9 ± 1.8*	409.4 ± 2.6*	512.3 ± 3.2*	682.3 ± 2.5*	741.8 ± 11.0*
	OZR + HYD	244.0 ± 3.9*	411.5 ± 3.4*	510.4 ± 9.2*	606.0 ± 4.8*^,†^	—
	OZR + CAP	239.0 ± 2.7*	409.0 ± 4.5*	515.3 ± 4.6*	624.3 ± 8.6*	—
	OZR + GEM	257.0 ± 4.3*^,†^	403.7 ± 3.6*	514.4 ± 4.7*	631.7 ± 5.8*^,†^	—
	OZR + ATOR	246.0 ± 1.7*^,†^	404.5 ± 3.2*	493.5 ± 10.0*^,†^	615.2 ± 5.4*^,†^	—
	OZR + MET	237.0 ± 1.0*	—	485.0 ± 6.1*^,†^	658.8 ± 4.2*^,†^	—
	OZR + ROSI	246.8 ± 2.9*	—	475.7 ± 3.1*^,†^	680.2 ± 5.1*	—
	OZR + TEM	233.0 ± 3.6*	412.0 ± 11.0*	525.5 ± 9.2*	612.0 ± 12.7*†	—
	OZR + LNM	238.5 ± 5.1*	407.8 ± 8.1*	501.0 ± 3.8*^,†^	597.3 ± 3.7*^,†^	—
Insulin (ng/ml)	LZR	1.0 ± 0.1^†^	1.2 ± 0.1^†^	1.3 ± 0.1^†^	1.1 ± 0.1^†^	1.5 ± 0.1^†^
	OZR	3.5 ± 0.1*	5.0 ± 0.1*	7.6 ± 0.2*	7.8 ± 0.1*	10.8 ± 0.6*
	OZR + HYD	3.9 ± 0.1*^,†^	5.5 ± 0.3*	8.1 ± 0.3*^,†^	9.5 ± 0.3*^,†^	—
	OZR + CAP	3.5 ± 0.2*	3.7 ± 0.3*^,†^	5.4 ± 0.3*^,†^	6.7 ± 0.2*^,†^	—
	OZR + GEM	4.4 ± 0.2*^,†^	5.7 ± 0.3*^,†^	7.3 ± 0.3*	8.6 ± 0.3*^,†^	—
	OZR + ATOR	3.7 ± 0.2*	4.4 ± 0.1*^,†^	5.7 ± 0.3*^,†^	6.3 ± 0.2*^,†^	—
	OZR + MET	3.4 ± 0.2*	—	4.3 ± 0.1*^,†^	5.3 ± 0.1*^,†^	—
	OZR + ROSI	3.5 ± 0.1*	—	4.0 ± 0.1*^,†^	5.1 ± 0.1*^,†^	—
	OZR + TEM	3.4 ± 0.3*	5.2 ± 0.3*	8.1 ± 0.5*	9.9 ± 0.3*^,†^	—
	OZR + LNM	3.9 ± 0.2*	5.8 ± 0.3*	9.9 ± 0.2*^,†^	11.3 ± 0.4*^,†^	—
Glucose (mg/dL)	LZR	93.7 ± 1.1^†^	98.4 ± 1.1^†^	100.9 ± 1.1^†^	100.2 ± 1.4^†^	104.7 ± 2.0^†^
	OZR	99.7 ± 1.4*	118.6 ± 3.4*	138.6 ± 2.7*	179.1 ± 1.2*	182.6 ± 2.7*
	OZR + HYD	101.0 ± 1.8*	104.5 ± 2.5*^,†^	134.2 ± 3.8*	170.5 ± 3.3*	—
	OZR + CAP	99.0 ± 3.5*	100.0 ± 2.5^†^	123.5 ± 2.5*^,†^	137.5 ± 5.3*^,†^	—
	OZR + GEM	100.9 ± 2.6*	105.2 ± 2.3*^,†^	126.0 ± 3.5*^,†^	170.2 ± 3.1*	—
	OZR + ATOR	96.2 ± 1.1*	109.8 ± 2.2*^,†^	128.7 ± 2.2*^,†^	159.1 ± 3.5*^,†^	—
	OZR + MET	123.7 ± 2.1*^,†^	—	121.8 ± 1.5*^,†^	132.5 ± 2.3*^,†^	—
	OZR + ROSI	121.5 ± 0.9*^,†^	—	118.0 ± 1.8*^,†^	126.8 ± 3.3*^,†^	—
	OZR + TEM	97.5 ± 4.1*	110.8 ± 10.9*	134.3 ± 5.5*	170.5 ± 4.5*	—
	OZR + LNM	95.5 ± 1.7*	123.3 ± 6.4*	149.5 ± 3.9*	174.8 ± 4.0*	—

*p < 0.05 vs. LZR at that age; †*p* < 0.05 vs. OZR at that age.

**TABLE 4:** Skeletal muscle vascular component data, presented as mean ± SE246, for the animal groups of the present study across all age.

Component	Group	7 wk	10 wk	13 wk	17 wk	20 wk
Acetylcholine dilation (μm)	LZR	119.2 ± 1.1	125.0 ± 1.1^†^	129.3 ± 1.2^†^	136.5 ± 2.0^†^	139.9 ± 1.6^†^
	OZR	115.3 ± 1.9	118.4 ± 3.2*	120.5 ± 2.8*	122.6 ± 2.9*	119.3 ± 3.3*
	OZR + HYD	117.6 ± 2.6	127.3 ± 2.2^†^	131.7 ± 2.3^†^	129.6 ± 3.4*	—
	OZR + CAP	118.3 ± 2.7	117.0 ± 2.1*	124.8 ± 3.9	124.3 ± 1.9*	—
	OZR + GEM	125.5 ± 3.1^†^	126.0 ± 1.8^†^	122.1 ± 2.9*	121.6 ± 2.9*	—
	OZR + ATOR	124.0 ± 3.4*^,†^	129.1 ± 2.7^†^	129.3 ± 2.9^†^	134.6 ± 2.7^†^	—
	OZR + TEM	113.8 ± 4.8*	120.5 ± 2.2	119.5 ± 5.6*	118.8 ± 5.4*	—
	OZR + LNM	99.3 ± 4.3*^,†^	99.25 ± 4.2*^,†^	109.5 ± 6.3*^,†^	108.5 ± 1.7*^,†^	—
Microvessel density (#/mm^2^)	LZR	809.9 ± 13.9	810.4 ± 13.1	811.8 ± 11.9†	863.1 ± 4.6^†^	823.0 ± 9.1^†^
	OZR	805.4 ± 25.2	782.4 ± 26.1	706.9 ± 16.9*	656.3 ± 20.5*	635.9 ± 11.6*
	OZR + HYD	881.4 ± 10.8*^,†^	874.6 ± 14.6*^,†^	832.3 ± 21.5^†^	869.4 ± 17.3^†^	—
	OZR + CAP	833.3 ± 12.3	813.3 ± 5.5	764.0 ± 8.0*^,†^	730.5 ± 16.6*^,†^	—
	OZR + GEM	830.2 ± 16.5	770.2 ± 12.8*	688.0 ± 11.6*	660.2 ± 10.9*	—
	OZR + ATOR	806.4 ± 8.4	803.4 ± 4.3	785.2 ± 5.0*^,†^	769.1 ± 7.1*^,†^	—
	OZR + TEM	839.0 ± 6.2*	841.4 ± 7.3*^,†^	850.2 ± 9.6*^,†^	814.1 ± 6.3*^,†^	—
	OZR + LNM	833.3 ± 7.8	798.5 ± 21.3	722.3 ± 12.0*	638.5 ± 8.3*^,†^	—
Stress vs. strain *β*	LZR	2.6 ± 0.1	2.6 ± 0.1^†^	2.8 ± 0.1^†^	3.1 ± 0.1^†^	3.2 ± 0.1^†^
	OZR	2.4 ± 0.3	3.4 ± 0.5*	4.1 ± 0.6*	6.2 ± 0.4*	5.7 ± 0.7*
	OZR + HYD	3.7 ± 0.3*	3.5 ± 0.3*	3.8 ± 0.4*	4.0 ± 0.3*^,†^	—
	OZR + CAP	3.2 ± 0.2*	3.5 ± 0.3*	4.2 ± 0.2*	4.8 ± 0.3*^,†^	—
	OZR + GEM	3.0 ± 0.1*	4.0 ± 0.2*	5.7 ± 0.3*^,†^	6.4 ± 0.1*	—
	OZR + ATOR	3.1 ± 0.1*^,†^	3.8 ± 0.3*	5.1 ± 0.2*	5.0 ± 0.3*^,†^	—
	OZR + TEM	3.4 ± 0.6*^,†^	3.8 ± 0.2*	4.4 ± 0.2*	6.3 ± 0.1*	—
	OZR + LNM	3.4 ± 0.2*^,†^	4.4 ± 0.2*^,†^	5.5 ± 0.3*^,†^	7.0 ± 0.2*^,†^	—

*p < 0.05 vs. LZR at that age; ^†^ *p* < 0.05 vs. OZR at that age.

**TABLE 5:** Cerebral vascular component data, presented as mean ± SE, for the animal groups of the present study across all age.

Component	Group	7 wk	10 wk	13 wk	17 wk	20 wk
Acetylcholine dilation (μm)	LZR	135.2 ± 1.3^†^	144.1 ± 0.9^†^	151.8 ± 0.8^†^	155.0 ± 1.4^†^	163.0 ± 1.1^†^
	OZR	122.4 ± 1.3*	128.4 ± 1.0*	125.1 ± 0.8*	122.9 ± 1.7*	119.8 ± 2.5*
	OZR + HYD	117.6 ± 2.6	—	131.7 ± 2.3^†^	129.6 ± 3.4*	—
	OZR + CAP	137.5 ± 0.7	—	139.0 ± 0.7*	132.5 ± 2.4*	—
	OZR + MET	133.2 ± 0.9^†^	—	134.2 ± 0.9*^,†^	132.3 ± 1.6*^,†^	—
	OZR + ROSI	134.6 ± 0.5^†^	—	135.7 ± 1.7*^,†^	127.5 ± 1.2*^,†^	—
	OZR + TEM	135.3 ± 0.3^†^	—	133.7 ± 1.6*^,†^	134.2 ± 1.6*^,†^	—
	OZR + LNM	126.2 ± 0.9*	—	117.3 ± 2.5*^,†^	112.8 ± 1.2*^,†^	—
Microvessel density (#/mm^2^)	LZR	290.0 ± 1.9^†^	293.0 ± 2.1^†^	303.8 ± 1.2^†^	313.6 ± 1.4^†^	319.6 ± 1.0^†^
	OZR	274.0 ± 3.2*	270.1 ± 2.6*	255.8 ± 2.6*	249.1 ± 2.1*	242.0 ± 1.9*
	OZR + HYD	341.0 ± 3.0*^,†^	—	308.3 ± 2.0^†^	279.7 ± 2.1*^,†^	—
	OZR + CAP	337.3 ± 2.7*^,†^	—	332.0 ± 1.7*^,†^	310.3 ± 2.2^†^	—
	OZR + MET	329.0 ± 2.4*^,†^	—	334.7 ± 3.2*^,†^	322.3 ± 2.3^†^	—
	OZR + ROSI	333.3 ± 2.5*^,†^	—	309.7 ± 3.4^†^	322.0 ± 2.6^†^	—
	OZR + TEM	341.7 ± 1.2*^,†^	—	341.0 ± 1.2*^,†^	333.3 ± 2.8^†^	—
	OZR + LNM	331.2 ± 1.6	—	305.0 ± 1.8^†^	257.3 ± 1.1*	—
Stress vs. strain *β*	LZR	1.6 ± 0.1	1.7 ± 0.1^†^	1.8 ± 0.1^†^	2.0 ± 0.1^†^	2.2 ± 0.1^†^
	OZR	1.8 ± 0.1	2.1 ± 0.1*	2.8 ± 0.2*	4.0 ± 0.2*	5.4 ± 0.1*
	OZR + HYD	3.7 ± 0.3*	—	3.8 ± 0.4*	4.0 ± 0.3*^,†^	—
	OZR + CAP	2.5 ± 0.1*^,†^	—	3.3 ± 0.1*^,†^	4.7 ± 0.1*^,†^	—
	OZR + MET	2.5 ± 0.1*^,†^	—	3.5 ± 0.1*^,†^	6.0 ± 0.1*^,†^	—
	OZR + ROSI	2.6 ± 0.1*^,†^	—	3.5 ± 0.1*^,†^	6.1 ± 0.1*^,†^	—
	OZR + TEM	2.5 ± 0.1*^,†^	—	3.4 ± 0.1*^,†^	5.6 ± 0.1*^,†^	—
	OZR + LNM	2.6 ± 0.1	—	4.5 ± 0.1*^,†^	7.1 ± 0.2*^,†^	—

*p < 0.05 vs. LZR at that age; ^†^ *p* < 0.05 vs. OZR at that age.

**FIGURE 2:**
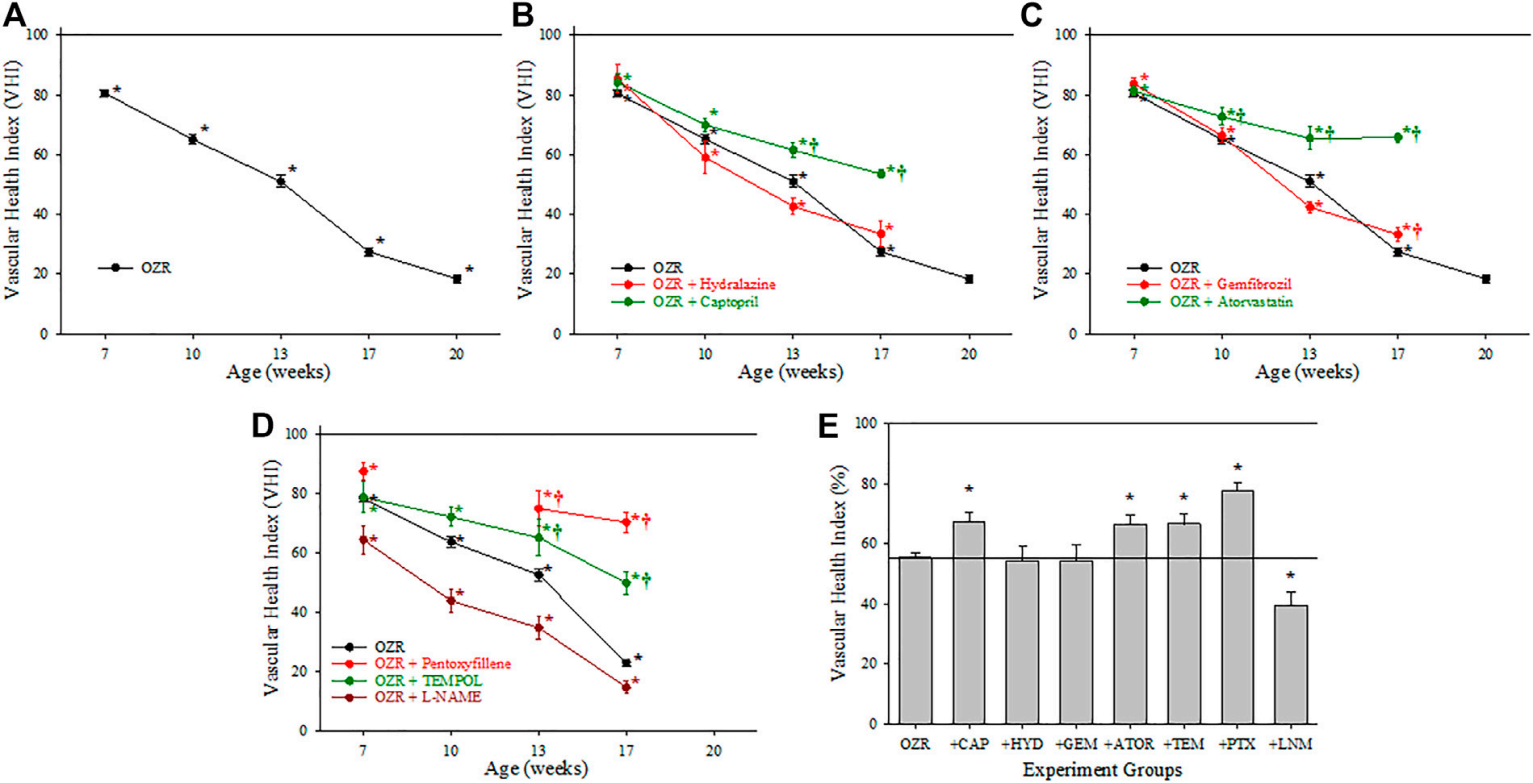
Data describing the three-parameter determination of Vascular Health Index (VHI) within the skeletal muscle microcirculation. Data (mean ± SE) are presented for OZR over the age ranges of the present study **(A)** or the impact of chronic anti-hypertensive **(B)**, anti-dyslipidemia **(C)**, or antioxidant/anti-inflammatory/nitric oxide bioavailability **(D)** therapies. **(E)** presents the aggregate VHI from the different animal groups where all ages have been compiled into one data point. By definition, VHI from LZR is set to 100%. *p < 0.05 vs. LZR at that age; ^†^ *p* < 0.05 vs. OZR at that age. Please see text for details.

Figure 2 summarizes the data across the conditions of the present study for the calculation of the VHI in the skeletal muscle vasculature. Panel A present the data from untreated OZR in relation to the healthy control, the untreated, age-matched LZR, where VHI fell steadily with increasing severity and duration of the metabolic disease. Treatment with the anti-hypertensive agents hydralazine or captopril (Panel B) resulted in differential effects in terms of skeletal muscle VHI, where captopril resulted in a consistent blunting of the reduction to VHI, while hydralazine was without consistent impact. Panel C presents the results on skeletal muscle VHI for OZR treated with the anti-dyslipidemia agents gemfibrozil and atorvastatin. Comparable to that for treatment with the anti-hypertensive agents, there was a divergence in outcomes, with atorvastatin blunting the impaired VHI and gemfibrozil having minimal impact. Panel D presents the impact of agents targeting chronic pro-oxidant (TEMPOL) and pro-inflammatory (pentoxifylline) environments in OZR. Both agents were highly effective at reducing the decline in skeletal muscle VHI across the age range of the present study. In addition, treatment of OZR with L-NAME (to remove nitric oxide bioavailability and accelerate the impact of chronic metabolic disease) increased the rate of decline in VHI across all ages of OZR. Panel E presents the time-averaged changes in VHI in OZR under the different interventions in the present study as compared to that for control LZR, and clearly demonstrates that treatment with captopril, atorvastatin, TEMPOL and pentoxifylline were most effective at improving vascular outcomes.

**FIGURE 3:**
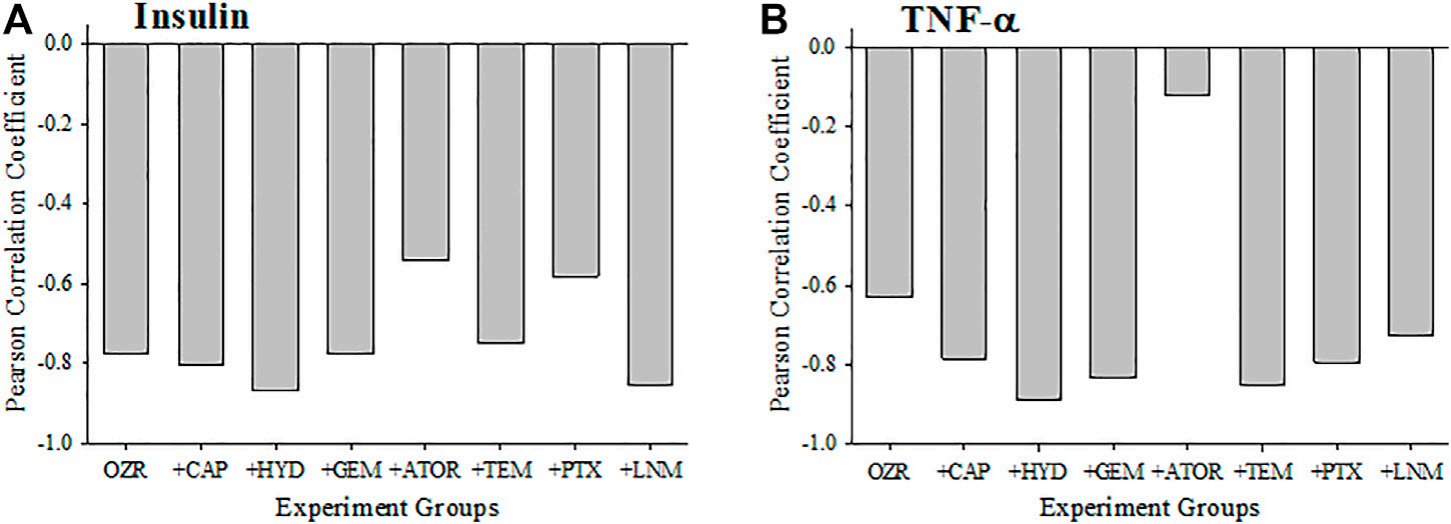
Data (mean ± SE) describing the criterion validity between plasma insulin **(A)** or TNF-α **(B)** and the skeletal muscle Vascular Health Index (VHI) across the different treatment groups in the present study. Criterion validity is demonstrated by a strong, negative Pearson Correlation coefficient between insulin or TNF-α and VHI in OZR. Please see text for details.

Figure 3 presents the criterion validity for the calculations of the peripheral VHI in the present study through determination of the Pearson Correlation Coefficients with plasma insulin and TNF-a levels; both well-established markers of metabolic disease severity. Both plasma insulin (Panel A) and TNF-a (Panel B) concentrations were strongly and negatively correlated with the VHI. This correlation reflects the strong tendency for OZR with healthier vasculature and higher VHI will have the lowest insulin resistance and chronic inflammation. The exception to this pattern is with the correlation between TNF-a and VHI under following treatment with atorvastatin, which was much weaker, indicating a much improved overall vascular health through the measured parameters.

**FIGURE 4:**
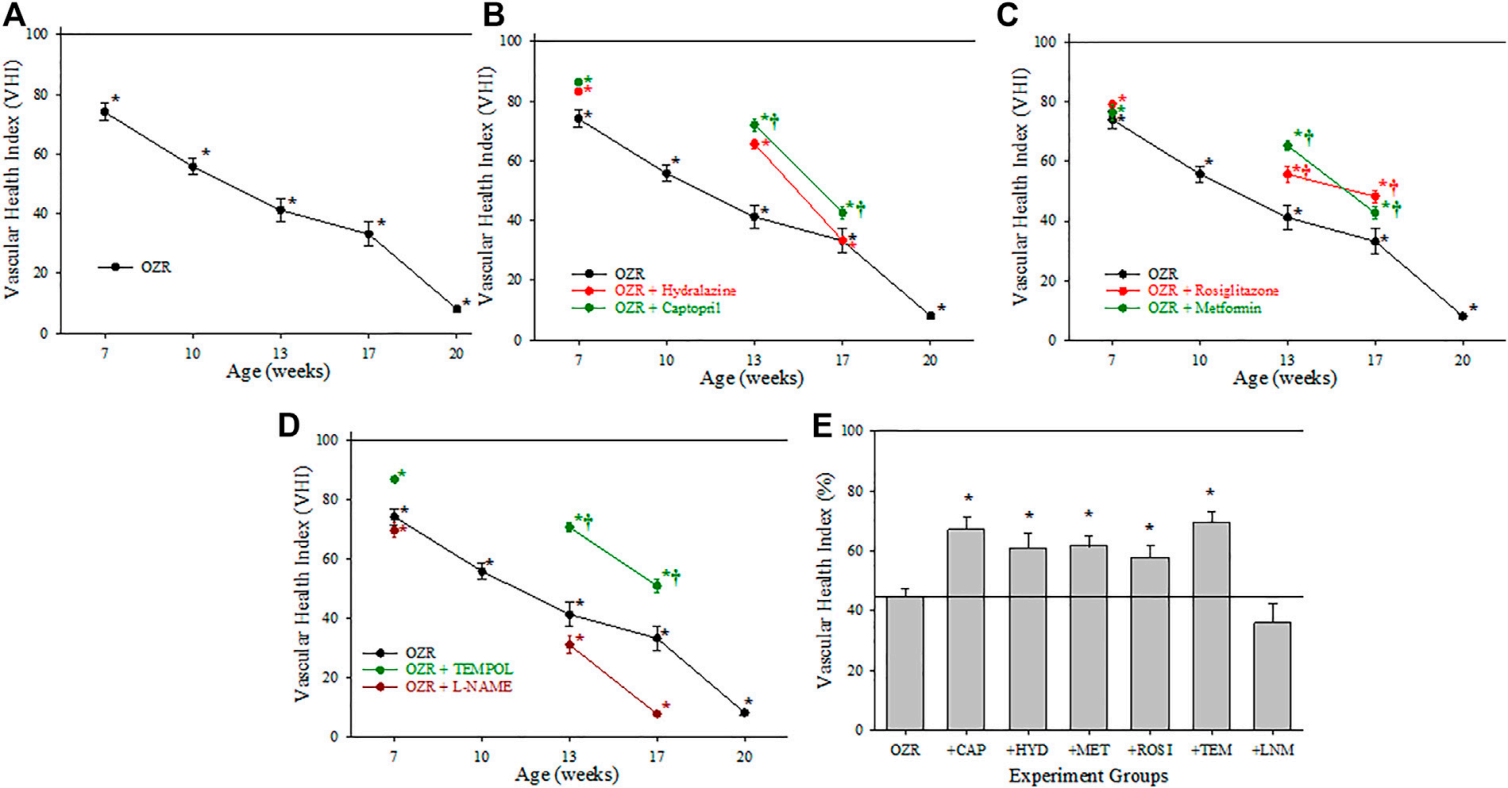
Data describing the three-parameter determination of Vascular Health Index (VHI) within the cerebral microcirculation. Data (mean ± SE) are presented for OZR over the age ranges of the present study **(A)** or the impact of chronic anti-hypertensive **(B)**, anti-diabetic **(C)**, or antioxidant/anti-inflammatory/nitric oxide bioavailability **(D)** therapies. **(E)** presents the aggregate VHI from the different animal groups where all ages have been compiled into one data point. By definition, VHI from LZR is set to 100%. *p < 0.05 vs. LZR at that age; ^†^ *p* < 0.05 vs. OZR at that age. Please see text for details.

Figure 4 presents the calculations of cerebral VHI in OZR under the conditions of the present study. Panel A presents the progressive deterioration in cerebral VHI in OZR versus LZR out to 20 weeks of age. Chronic treatment with the antihypertensive agents (Panel B) or with the anti-diabetic agents (to improve glycemic control, Panel C) resulted in temporary improvements to cerebral VHI, although the results suggest that this effect may be insufficient to maintain VHI over time. A similar general pattern was determined for cerebral VHI in OZR following treatment with the antioxidant TEMPOL (Panel D), where beneficial effects on vascular health appeared to decay with increasing severity and duration of metabolic disease. Reducing nitric oxide bioavailability with L-NAME resulted in significant reductions to VHI in comparison to untreated OZR, accelerating the progression of cerebrovasculopathy. The time-averaged VHI for the cerebral circulation of OZR under the conditions of the present study are summarized in Panel E, where the employed interventions improved the aggregate VHI as compared to that in OZR.

**FIGURE 5:**
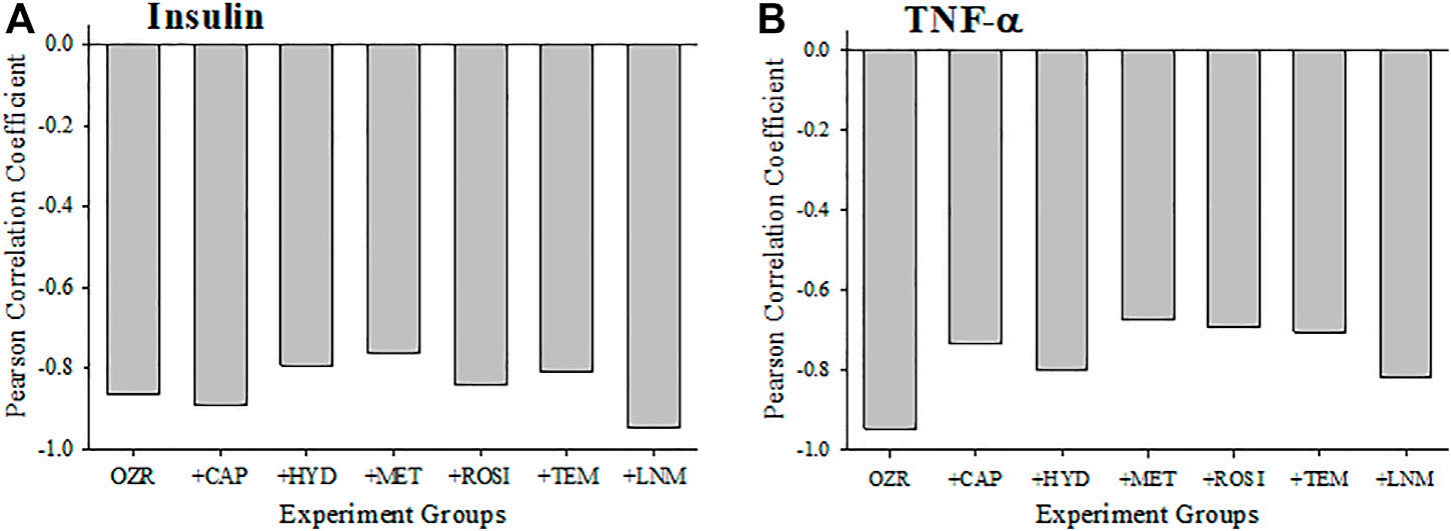
Data (mean ± SE) describing the criterion validity between plasma insulin **(A)** or TNF-α **(B)** and the cerebral Vascular Health Index (VHI) across the different treatment groups in the present study. Criterion validity is demonstrated by a strong, negative Pearson Correlation coefficient between insulin or TNF-α and VHI in OZR. Please see text for details.

The criterion validity for the calculations of cerebral VHI in the present study with plasma insulin and TNF-a levels are presented in Figure 5. Both plasma insulin (Panel A) and TNF-a (Panel B) concentrations were strongly and negatively correlated with the VHI and this was demonstrated across all interventions. Comparable to the interpretation for skeletal muscle VHI, these results indicate the strong correlation between healthier vasculature and higher VHI with low levels of insulin resistance and chronic inflammation in the OZR cerebrovasculature.

## Discussion

This study describes the approach for developing and using a vascular health index (VHI) representing integrated vascular function while allowing for the pooling of data across many different studies to maximize inferential power and available analytic approaches. We did this by using our raw data from multiple studies to calculate this new metric over the temporal development of metabolic disease in OZR and quantify the associated progressive dysfunction within the microcirculation. Additionally, we used our metric to outline the impact of three major classes of clinically relevant pharmacological interventions (anti-hypertension, anti-dyslipidemia, anti-diabetes) along with antioxidant treatment and present the relative effectiveness of each intervention in improving integrated microvascular health within the OZR manifesting metabolic disease. Throughout this process, we demonstrated the face, content, criterion and discriminant validity of the VHI; suggesting that this metric of integrated vascular function can be used for more complex analyses moving forward.

The development of the VHI has several characteristics that deserve some comment. Foremost, it is not intended to be a predictive score [such as things like the Framingham Risk Score (Cooper et al., 2020; Sumayin Ngamdu et al., 2020)], and as such we have included no parameter weighting in its calculation. This is important to note as there is no clear *a priori* rationale justifying parameter weighting at this time and there is also no consensus regarding the relative importance of vascular reactivity, wall mechanics or microvessel density in terms of contributions to health outcomes. Finally, parameter weighting, by definition, requires the use of a regression-based approach designed to predict an outcome. As this was not the purpose of VHI, this was rejected in favor of the analytical approach employed (Menon et al., 2022).

As presented Figure 1, the calculation of either skeletal muscle or cerebral VHI was dependent on the integration of three parameters, acetylcholine-induced dilation (as a marker of overall reactivity and endothelial function), the slope of the circumferential stress vs. strain relation of the vascular wall (as a marker of vascular wall stiffness and overall mechanics), and microvessel density (MVD; as a central determinant of the ability of the microvascular networks to effectively deliver and exchange materials to metabolic active tissues). Each of these three markers has been well documented in the existing literature to be highly predictive of a poor vascular outcomes and the impact of cardio/cerebrovascular disease risk (Chu et al., 2001; Silveira Rossi et al., 2022). While other markers of vascular structure and function could certainly be used if desired by an investigative group to assess a different outcome, we believe that these three markers are not only relatively straightforward to determine and do cover the major aspects of vascular/microvascular health (Effros et al., 1981). Thorough discussions of the mechanistic bases of the impact of chronic metabolic disease and the pharmacological interventions employed in the present study have been presented elsewhere and will not be covered in detail in this manuscript.

In terms of demonstrating the validity of the skeletal muscle and cerebral VHI, it is important to note that two plasma biomarkers that have been well-established indicators of chronic metabolic disease severity and duration, plasma insulin (Vogelzangs et al., 2020; Koenen et al., 2021) and TNF-α (Barrett et al., 2017; Koenen et al., 2021) concentrations, demonstrated strong negative correlations with VHI in untreated OZR. Following the imposition of multiple pharmacological interventions used in this study, there was some reduction to the correlation coefficient, suggesting the creation of a healthier vasculature, although the clear correlations still remained, as would be expected.

In terms of discriminant validity for VHI, much of this is provided by the changes in the individual parameters comprising the index (vascular reactivity, vascular wall mechanics and microvessel density) in response to the most effective pharmacological interventions (e.g., atorvastatin and rosiglitazone). However, the use of L-NAME in OZR deserves a brief discussion. L-NAME essentially eliminates vascular nitric oxide bioavailability and treating animals with it chronically can be considered to be a proxy for mimicking that impact of chronic metabolic disease on vascular health, albeit and accelerated one (Frisbee, 2005c). The more rapid decay in VHI in OZR with chronic L-NAME treatment is a clear demonstration of the ability of VHI to effectively discriminate between experimental conditions and different states of vascular health.

One of the most interesting aspects of the results of the present study is in regard to the timing and aggressiveness of pharmacological interventions to maintain skeletal muscle and cerebral vascular health in chronic metabolic disease. The results of the present study strongly suggest that aggressive prodromic intervention with appropriate pharmacological agents for treating risk factors such as hypertension, impaired glycemic control, atherogenic dyslipidemia and the pro-oxidant and pro-inflammatory environments that are associated with them, may be most appropriate and effective in terms of maintaining vascular health over time. From the perspective of healthcare delivery, while it may be more effective in terms of patient outcomes to intervene earlier with a aggressive pharmacological avenues to maintain vascular health, especially given challenges in patient adherence and compliance with lifestyle changes, an aggressive early intervention may also be more economically sustainable given the exorbitant costs associated with chronic disease and end-organ damage if treatment is delayed (Janssen et al., 2020; Rangaswami et al., 2020).

Continuing with this concept, the results of the present study also suggested pharmacological agents targeted at one risk factor (e.g., metformin or rosiglitazone) may not be adequately effective in the setting of more complicated disease risk, demonstrating a progressive inability to maintain vascular health. These results suggest that multiple treatments may be required for realizing optimal outcomes, or that the benefits derived from pharmacological agents with pleiotropic effects such as atorvastatin (Frisbee et al., 2018), and to some extent anti-hypertensive agents (Frisbee et al., 2018), may allow for a greater maintenance of vascular health beyond that which would be expected from simple risk factor reduction alone. Recent study involving machine learning approaches have suggested these outcomes may be realized in chronic metabolic disease, where chronic interventions with pharmacological agents with beneficial impacts in addition to risk factor reduction were highly effective at improving functional outcomes that may not have been associated with the intended use of the drug (Nowak et al., 2022).

A key part of proposing a novel metric assessing the relative status of a physiological system is outlining meaningful applications (Menon et al., 2022). The demonstrated development and use of VHI allow for simple and accurate interpretations of the relative vascular health profiles and significant differences in integrated vascular health outcomes between animal models under different treatment regimes. Consequently, the development and evaluation of new pharmacological interventions addressing traditional cardiovascular disease risk factors like impaired lipid control and hypertension can benefit from a metric like VHI to guide and determine intervention’s associated anti-vasculopathy capabilities. Furthermore, VHI can also be used in the study of progressing preclinical symptoms and states of low CVD risk factors to elevated CVD risk factors and actual disease diagnoses to help determine optimal windows of treatment for different interventions to achieve ideal health outcomes. Finally, the described use of VHI allows us to link datasets across time, research groups and different studies to make such assessments and comparisons of interventions and their outcomes on vascular health more accessible and manageable. The effective use of tools such as VHI can assist investigators in better understanding of the role of vascular dysfunction in disease etiology and in assessments of the effectiveness of various interventions in addressing progressive vasculopathy.

## Data Availability

The data analyzed in this study is subject to the following licenses/restrictions: The datasets are currently in use for ongoing analyses and will not be made available until that has been completed. Summary data are presented in tabular form in the manuscript. Requests to access these datasets should be directed to jfrisbee@uwo.ca.
